# Soret Fishnet Metalens Antenna

**DOI:** 10.1038/srep09988

**Published:** 2015-05-07

**Authors:** Bakhtiyar Orazbayev, Miguel Beruete, Víctor Pacheco-Peña, Gonzalo Crespo, Jorge Teniente, Miguel Navarro-Cía

**Affiliations:** 1 Antennas Group-TERALAB, Universidad Pública de Navarra, Pamplona 31006, Spain; 2 Optical and Semiconductor Devices Group, Department of Electrical and Electronic Engineering, Imperial College London, London, SW7 2BT, UK; 3 Centre for Plasmonics and Metamaterials, Imperial College London, London, SW7 2AZ, UK; 4 Centre for Terahertz Science and Engineering, Imperial College London, London, SW7 2AZ, UK

## Abstract

At the expense of frequency narrowing, binary amplitude-only diffractive optical elements emulate refractive lenses without the need of large profiles. Unfortunately, they also present larger Fresnel reflection loss than conventional lenses. This is usually tackled by implementing unattractive cumbersome designs. Here we demonstrate that simplicity is not at odds with performance and we show how the fishnet metamaterial can improve the radiation pattern of a Soret lens. The building block of this advanced Soret lens is the fishnet metamaterial operating in the near-zero refractive index regime with one of the edge layers designed with alternating opaque and transparent concentric rings made of subwavelength holes. The hybrid Soret fishnet metalens retains all the merits of classical Soret lenses such as low profile, low cost and ease of manufacturing. It is designed for the W-band of the millimeter-waves range with a subwavelength focal length *FL* = 1.58 mm (0.5λ_0_) aiming at a compact antenna or radar systems. The focal properties of the lens along with its radiation characteristics in a lens antenna configuration have been studied numerically and confirmed experimentally, showing a gain improvement of ~2 dB with respect to a fishnet Soret lens without the fishnet metamaterial.

The Soret lens is a member of the Fresnel-zone plate lens (FZPL) family consisting of alternating transparent and opaque concentric rings^1^. In his seminal paper, written more than a century ago, Soret devised this lens as a purely optical device. Ultimately, the same concept has been successfully applied throughout the whole electromagnetic spectrum and in particular in the microwave range for lens antennas^2^,^3^,^4^. At microwaves these lenses present interesting characteristics such as low profile, low cost and ease of fabrication.

The main drawback of FZPL for microwave applications is a low efficiency in transmission compared to conventional lenses, due to high reflections from opaque zones. For antenna applications, such as Fresnel-zone plate antennas (FZPA), the low focusing efficiency deprecates the radiation efficiency, making them less competitive than other conventional antennas. Also the best performance of FZPA is obtained with large focal distance/diameter ratio (*F*/*D* ~ 3-15λ_0_)^3^. Thus, the volume enclosing the Soret lens along with the feeder is very large, making FZPA not suitable for compact systems. This leaves original FZPL and FZPA attractive only for few niches such as broadcast satellite reception^3^.

An advanced design of FZPL and FZPA reduces the amount of reflected power, i.e. improves the efficiency, by introducing selective phase shifts instead of blocking negative (odd) or positive (even) Fresnel zones^3^,^4^. This smart approach leads to high gain, high aperture efficiency and low side lobe level^5^. However, this increases the thickness and complicates the design and, as a result, the fabrication and cost.

Another possible way to improve the performance of the FZPA is to replace the transparent rings of the Soret lens by subwavelength hole arrays, which allows the excitation of surface currents (leaky-waves) and could improve coupling-in and -out of the spatial wave as it happens in the extraordinary transmission phenomenon^6^. Moreover, by introducing at the input of the FZPA a metamaterial slab with near zero index (NZIM) it is possible to increase the directivity of the antenna and reduce the sidelobe level, since the phase front at the output surface of the NZIM follows its shape and therefore the electromagnetic field becomes locally closer to a plane-wave distribution^7^,^8^,^9^. Recently, the fishnet metamaterial (i.e. closely-packed subwavelength hole arrays working under extraordinary optical transmission^6^,^10^,^11^ and emulating negative refractive index media and NZIM) was proposed for advanced lens design^12^,^13^,^14^. Their performance in the millimetre-wave regime has been confirmed in previous works^12^,^13^,^14^,^15^,^16^,^17^. Alike the single holey plate exhibiting extraordinary transmission, the leaky-wave mechanism present in the fishnet metamaterial may facilitate the coupling-in and –out, guaranteeing then a low insertion loss^11^, and may improve the effective illumination and extend the radiating area^18^, increasing the total efficiency of the lens antenna. This increased illumination efficiency allows a comparatively smaller *F*/*D* ratio, which means that the volume of FZPA can be reduced, by placing the transmitter closer to the FZPL. Another advantage of the fishnet metamaterial is the possibility to tailor an effective impedance matched with the free space, in the desirable NZIM regime.

In this work we propose a short focal length (*FL*) ultrathin hybrid Soret fishnet metalens with enhanced adaptation and antenna effective area. This hybrid design combines the advantages of the fishnet metamaterial and the Soret lens to overcome each other's drawbacks. The proposed Soret fishnet metalens has been designed, fabricated and experimentally analysed at the W-band of the millimetre-waves range. The experimental results show a gain of 10.64 dB at the operation frequency *f*_0_ = 96.45 GHz (λ_0_ = 3.11 mm). The experimental radiation patterns are supported by numerical results.

## Design procedure

### Fishnet metamaterial dispersion

**Figure 1:**
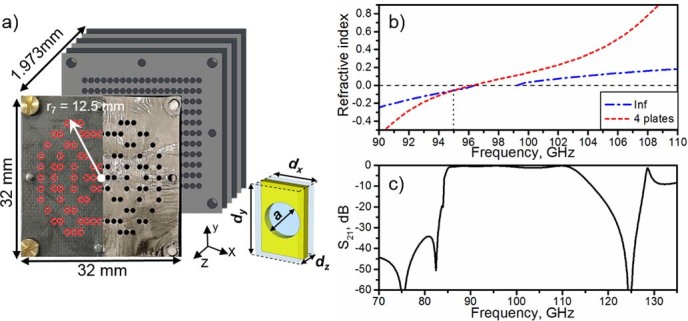
The hybrid Soret fishnet metamaterial lens. (a) Fabricated Soret fishnet metamaterial lens and its design with seven Fresnel zones. Light grey slabs account for perforated plates and dark grey for RO5880™ slabs. Fishnet metamaterial unit cell (Inset); (b) Effective refractive index, *n_m_*, for a fishnet metamaterial made of four (red dashed curve) and an infinite number of plates (blue dash dotted curve); (c) Transmission coefficient for a four-plate fishnet metamaterial.

The unit cell of the fishnet metamaterial used in this work (inset of Fig. 1(a)) is designed using the commercial substrate Rogers RO5880™ and has the following dimensions: *d_x_* = 1.26 mm, *d_y_* = 2.1 mm, *d_z_* = 0.398 mm (metal thickness *w* = 0.017 mm and thickness of dielectric *t_d_* = 0.381 mm), hole diameter *a* = *d_y_*/4 = 0.525 mm and spacer dielectric permittivity *ε_r_* = 2.2, with loss tangent tanδ = 9×10^−4^. For these parameters, the cut-off frequency of the hole fundamental TE_11 _mode is 112 GHz. The dispersion diagram of the infinite structure is numerically calculated using the commercial software CST Microwave Studio^TM ^(see *Methods* for details). The first band appears at 87–96 GHz, as shown by the blue dash-dotted line in Fig. 1(b), and corresponds to the fishnet extraordinary transmission band^10^,^19^. However, the calculation for a finite number of plates (see *Methods* for details) deviates from the infinite structure curve due to the inhomogeneity of the fishnet metamaterial^20^. From our previous experience we found that four plates is a good trade-off between total thickness and electromagnetic performance in terms of insertion loss^12^,^13^,^14^,^15^,^16^,^17^,^21^. The effective index of refraction for 4 cascaded plates (see *Methods* for details) is shown in Fig. 1(b) (dashed red line). We notice that the bandgap between 97 and 99 GHz due to Wood’s anomaly disappears in this case. This is more evident in the transmission coefficient *S_21_* for 4 cascaded plates, shown in Fig. 1(c). This feature has been observed before and is due to the tunneling of energy when the number of plates is small^22^. With these results and bearing in mind that we want the fishnet metamaterial to behave as NZIM, we chose 95 GHz (λ_0 _~ 3.16 mm) as design frequency where the index of refraction is *n_m_* = −0.06.

### Soret lens design

The radii of the Fresnel zones *r_i_* of the Soret lens can be calculated using the next design equation^2^,^3^,^4^: 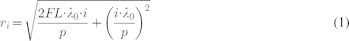
where *FL* is the focal length of the FZPL, λ_0_ is the operation wavelength, *i* is an integer denoting zone number and *p* is the number of phase quantization levels. Given the comparable size of the fishnet in-plane periods *d_x_* and *d_y_* with the radii of the Fresnel zones, the performance of the Soret fishnet metalens depends on the filling ratio of the Fresnel zones. This, in turn, depends on many parameters of the design, such as *FL*, λ, *i*, *p*. For this reason, to quickly and easily estimate the performance of the FZPL, a three-dimensional analytical calculation was done by means of the Huygens-Fresnel principle (See *Methods*). In this work the following parameters were chosen: *FL* = 1.58 mm (~0.5λ_0_), *i* = 7, *p* = 2. The final lens profile and fabricated Soret fishnet metalens is shown in Fig. 1(a). Unlike traditional designs, a subwavelength focal length *FL* = 0.5λ_0_ = 1.58 mm was deliberately chosen in this work to demonstrate the possibility of designing very compact systems. In order to improve the aperture efficiency, or equivalently the illumination efficiency, which is low due to the small focal length, an optimization routine was run, which included the numerical calculations of the directivity for the whole structure. A small number of Fresnel zones *i* = 7 was found to be optimal. A small number of phase quantization levels *p* = 2 was chosen for a better profile approximation, taking into account the finite periods *d_x_*, *d_y_* of the fishnet metamaterial. The radius of the last zone was *r_7_* = 12.5 mm. The positive (odd) zones were made of holes of the same dimension as the fishnet (see Fig. 1(a)). The final lens had a total thickness of 5*t_d_*+ 4*w* = 1.973 mm (~0.62λ_0_). Thus, the whole structure has dimensions of 32 mm × 32 mm × 1.973 mm.

## Results and discussion

### Analytical calculations for the Soret lens

**Figure 2:**
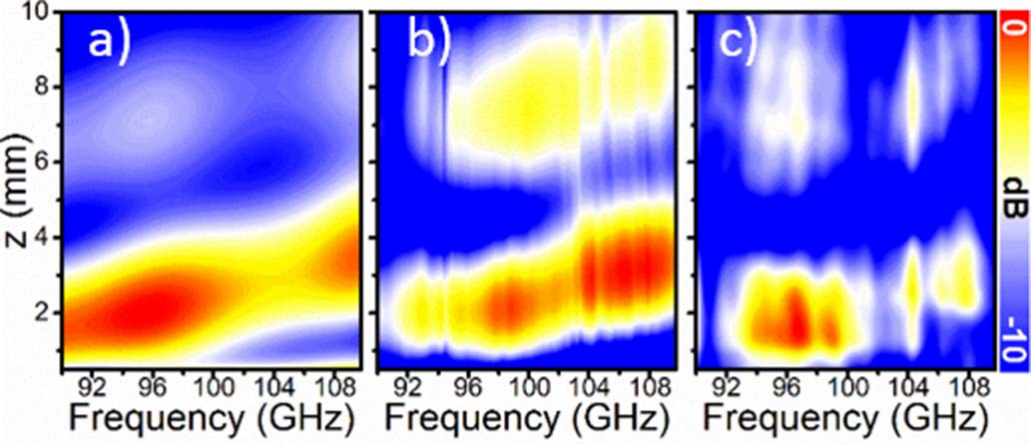
Power spectra along the optical axis. Power distribution along *z* axis for the frequency range 90–110 GHz: (a) analytical results, (b) simulation and (c) experimental results.

**Figure 3:**
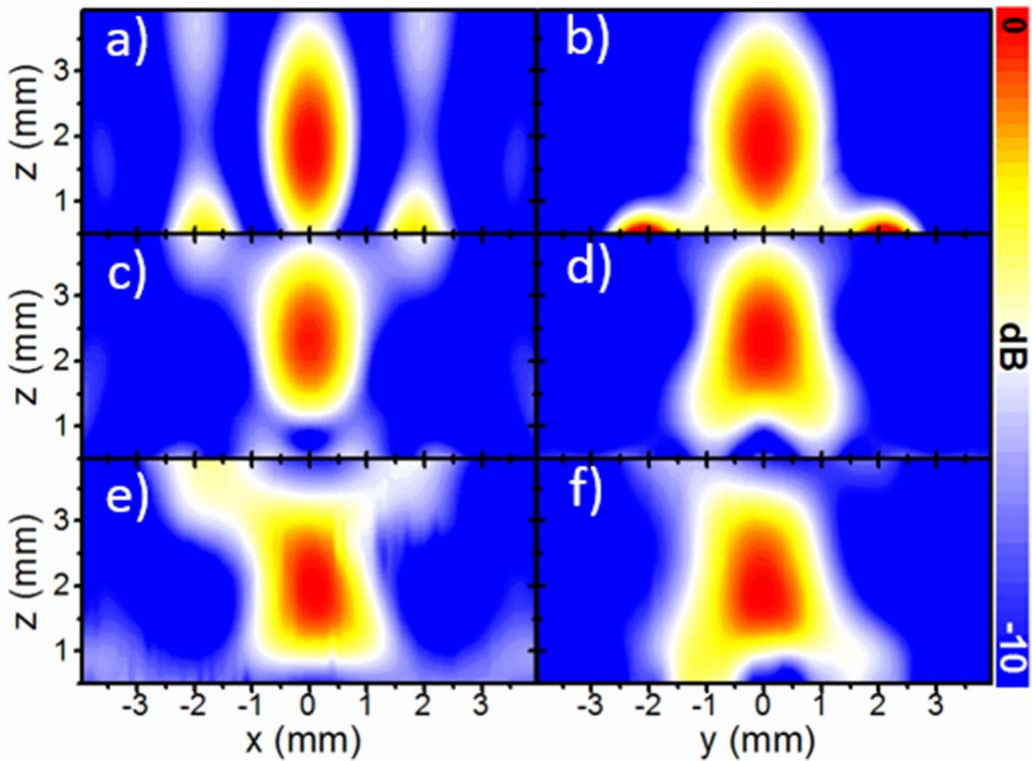
The normalized spatial power distribution on the *xz*-plane (left column) and *yz*-plane (right column) for: (a-b) analytical results at 95.5 GHz; (c-d) simulation results at 98.75 GHz; (e-f) experimental results at 96.45 GHz.

First, the power distribution was calculated as a function of frequency and position along the optical axis of the lens (Fig. 2(a)) using the Huygens-Fresnel principle. The absolute maximum in this case occurs at 95.5 GHz, with focal length *FL_1_* = 1.87 mm (~0.60λ_0_). The secondary focus is located at *FL_2_* = 7.01 mm (~2.22λ_0_) and has a significantly lower magnitude. The focal point shift with frequency evidences that the Soret lens suffers from chromatic aberration. Next, the power distribution colour-maps calculated at the design frequency are presented in Fig. 3(a, b) for the *xz*- and *yz*-plane respectively. In both cut-planes, clear foci can be observed with transverse dimensions 0.36λ_0_ and 0.5λ_0_ for *xz*- and *yz*-plane respectively.

### Simulation results for the Soret lens

With the design obtained after a fast prototyping with the Huygens-Fresnel principle, a full-wave numerical analysis of the realistic 3D model of the Soret fishnet metalens was done using the transient solver of the commercial software CST Microwave Studio™ (see *Methods*). These full-wave numerical results should provide a better modelling of the lens than the analytical results^23^, where some simplifications were made.

Initially, the power distribution as a function of frequency and position along the optical axis (*z*-axis) was obtained and is shown in Fig. 2(b). A focal spot appears within the frequency range 97–110 GHz, moving from 1.9 mm (0.57λ_0_) up to 3 mm (0.95λ_0_) along the *z*-axis. One can see that these results resemble closely the analytical results previously shown, but here the secondary focal spot is more prominent. The power enhancement (i.e., the ratio between the intensity with and without lens for each *xz* position) corresponding to the first maximum is 10.28 dB at 98.75 GHz (λ_0 _~ 3.04 mm) with a focal distance *FL* = 1.94 mm (~0.64λ_0_).

Finally, the colour-map of power distribution was generated for *xz*- and *yz-*planes at 98.75 GHz. These colour-maps resemble the analytical results but again show minor dissimilarities because of the different accuracy of each method. The blueshift of the focal spot can be simply explained however by taking into account that in the analytical calculations we neglect the dielectric substrate covering the first layer which changes the effective permittivity at the output surface.

### Experimental results for the Soret lens

**Figure 4:**
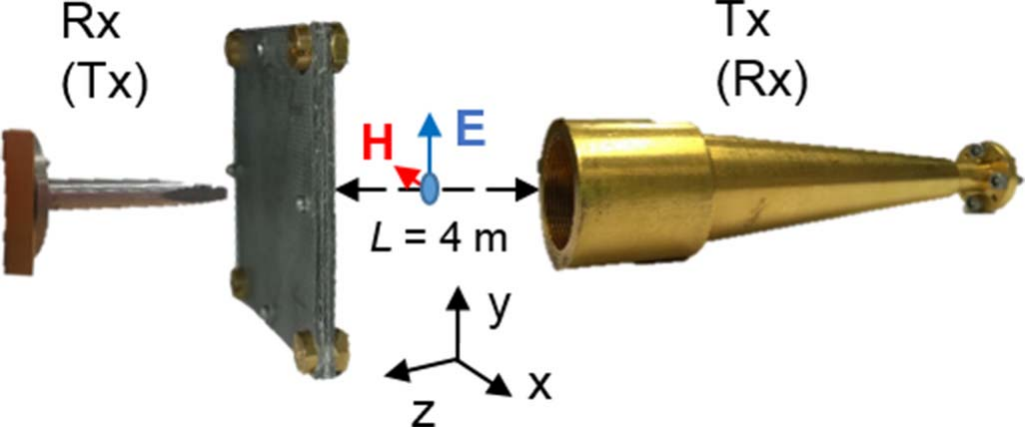
Experimental setup. Scheme of experimental setup for the focal measurements (radiation measurements) with a waveguide probe as a receiver (transmitter) and a standard high gain horn antenna as a transmitter (receiver).

Similarly to analytical and simulation results, colour-maps for the power spectrum along the optical *z*-axis and for the *xz*-, *yz*-planes were generated experimentally for the fabricated prototype, see Fig. 4 (details of the experiment can be found in *Methods*). The colour-map of the power distribution as a function of frequency and *z* position is shown in Fig. 2(c) and confirms our preliminary analytical and numerical results (Fig. 2(a,b)). The maximum power enhancement is 11.04 dB at 96.45 GHz (λ_0 _~ 3.11 mm) with a focal distance *FL_1_* = 1.9 mm (~0.61λ_0_). A secondary focus appears at *FL_2_* = 7 mm (2.25λ_0_) similarly to the analytical and numerical results. One can notice that for the experimental results the power enhancement is significantly lower within the frequency range 100–104 GHz. This can be due to the presence of thin air gaps between metallic and dielectric plates arising from imperfections of the fabrication. Additional simulations for the unit cell of the fishnet metamaterial were run and showed that indeed the air gaps inside the fishnet metamaterial deprecate its electromagnetic performance, reducing the transmitted power in this frequency range.

Next, the power was scanned on *xz*- and *yz*- planes at 96.45 GHz. The results of the spatial power distribution are shown in Fig. 3(e,f). The qualitative agreement with the numerical results is evident. To facilitate the comparison, all the results are gathered in Table I.

### Radiation properties: lens antenna

**Figure 5:**
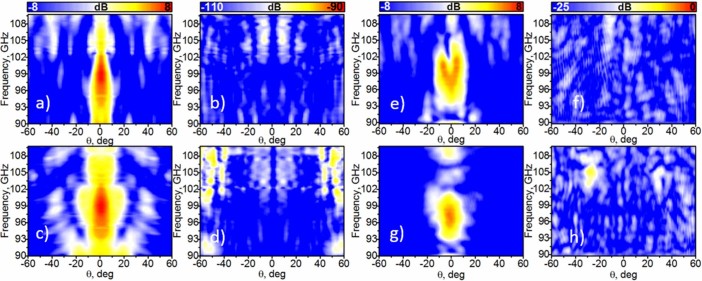
Radiation pattern vs. frequency. The numerical (a-d) experimental (e-h) radiation pattern of the Soret fishnet metamaterial lens antenna in the frequency range 90–110 GHz: co- (a, c, e, g) and cross- polarization (b, d, f, h). (a, b ,e, f) E-plane and (c, d, g, h) H-plane.

After a characterization of the focal properties of the Soret fishnet metalens, next we investigate its performance as FZPA (see *Methods*). Notice that now the input part of the hybrid lens is the Soret lens and the output is composed of the fishnet metamaterial. Numerical results of co- and cross-polar angular power distributions for *E-* and *H*-plane are displayed in Fig. 5(a-d). The maximum is located at 98.75 GHz, in agreement with the previous numerical study. It is evident from these figures that the angular beamwidth for *H*-plane is wider than for *E*-plane. This can be explained by the excitation of the leaky waves on the surface of the Soret lens, where the current density is higher and runs parallel to the *E*-plane^24^,^25^.

**Figure 6:**
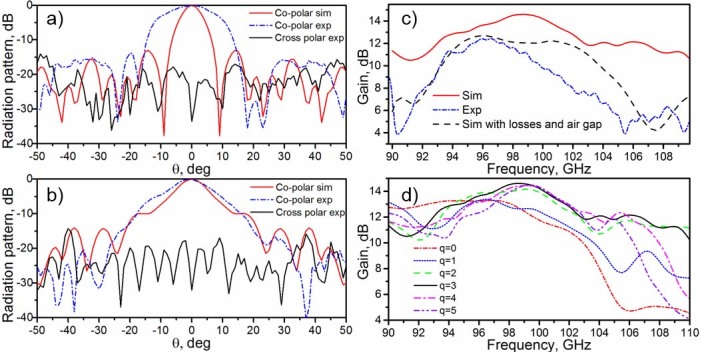
Normalized radiation pattern and gain. Normalized radiation patterns for: (a) E-plane and (b) H-plane at frequencies 98.75 GHz 96.45 GHz (simulation and experimental respectively). (c) The simulation gain (solid red line), the experimental gain (dash dotted blue line) and the simulation gain, considering air gaps and losses (dashed black line). (d) Simulation results for the gain of the Soret lens antenna with a different number of stacked plates *q*.

The normalized experimental results (see *Methods* for details about this experiment) for co- and cross-polar components as a function of frequency and angle are shown in Fig. 5(e-f) *E*-plane and Fig. 5(g-h) *H*-plane. One can notice the small disagreement in the co-polar component for *E*-plane, in particular the separation of the main beam. Even though the experiment was done with the greatest possible care and precision, this could be well explained by an undesired tilt of the lens in the experimental setup since additional simulations for a tilted lens (with a tilt angle θ = 7 deg in *H*-plane) were run and demonstrated a similar pattern (not shown here). Consistently with the previous experiment (investigating the focal properties of the Soret lens) the experimental maximum is located at 96.45 GHz, i.e. slightly shifted from the frequency obtained by simulation. In Fig. 6(a, b), we plot together numerical and experimental results of the maximum at each respective frequency. In this figure, to facilitate the comparison, the normalized simulation and experimental radiation patterns for the *E*- and *H*-plane are presented. Logically, the performance is different, and most notably the beamwidth is wider in the experiment. Additional simulations prove that the frequency shift of 2 GHz provokes broadening of the beamwidth from 8.7 deg up to 13 deg. Another factor for the wider beamwidth in the experimental *E*-plane is a displacement of the waveguide probe. For example, in our case the focal displacement of 0.3 mm in the simulations increases the beamwidth from 8.7 deg up to 11 deg. The combination of these two factors widens the beam more than two times – from 8.7 deg up to 18.5 deg.

To complete the study, the numerical and experimental gain for the Soret fishnet metamaterial lens antenna is presented in Fig. 6(c). In the experiment, the gain was obtained by comparing our lens antenna with a standard horn antenna following the gain comparison method^26^ (see *Methods*). A high gain of 10.64 dB is found experimentally at 96.45 GHz (solid blue line). The numerical value of 14.6 dB is found at frequency 98.75 GHz by using the software-implemented far-field monitors (dashed red line). The difference in the results, the lower values of gain and the shift in frequency, can be explained as a sum of all previously described factors, such as experimental errors (misalignment, accuracy of distance measurement) and by defects in the fabrication (non-perfect contact between dielectric and metallic plates) and effective substrate losses higher than nominal values. To confirm these factors, additional simulations for the complete 3D model of the FZPA were run with an air gap of 50 μm and higher dielectric loss tangent tanδ = 0.015. The resulting gain is shown in Fig. 6(c) (dashed dotted black line). Here an air gap of 50 μm between metallic and dielectric plates results in a frequency shift of the maximum from 98.75 GHz to 96.25 GHz and the higher losses result in the lower gain. Consequently, the numerical gain of the lens with higher losses and spurious air gap is closer to the experimental gain. The maximum directivity, computed readily from the simulation, is 26.0 dBi at 98.75 GHz, and a directivity 22.2 dBi is estimated from experimental results at 96.45 GHz^27^. To facilitate the comparison, all the results are gathered in Table I.

**Figure 7:**
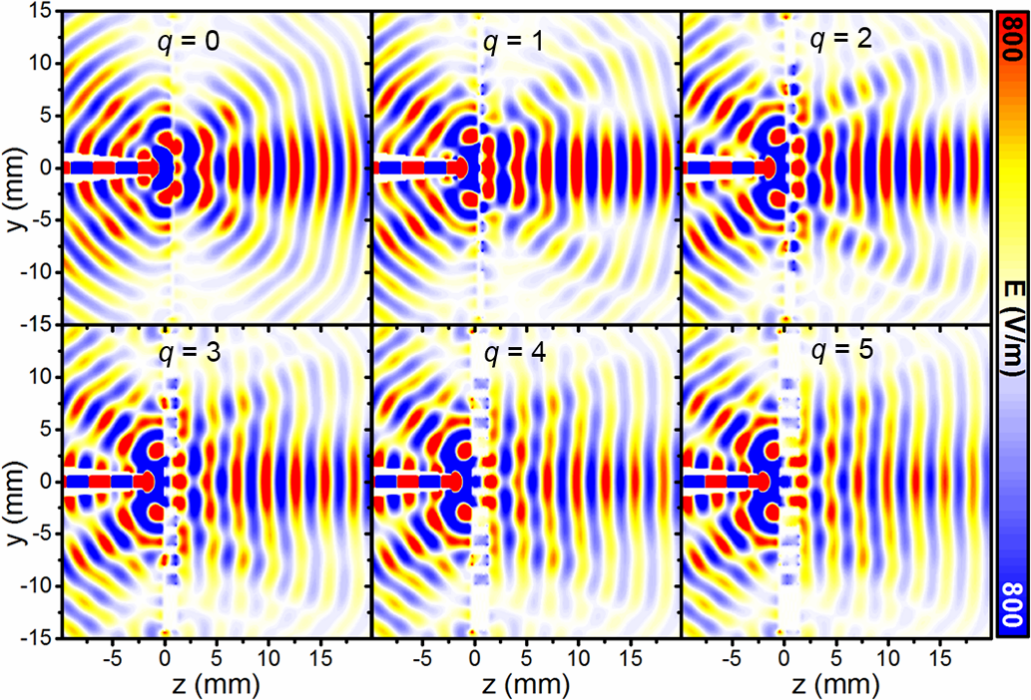
Electric field distribution on the *yz*-plane. The colour-maps for the distribution of *E_y_*component on the *yz*-plane for different number of stacked plates *q.*

In order to demonstrate the advantages and improvements of the hybrid Soret fishnet metamaterial lens antenna, we compare in this section its performance in terms of gain for an increasing number of cascaded fishnet plates. As it was mentioned in the introduction, NZIM can improve the radiation parameters due to the redistribution of the energy on its boundaries. Since the phase advance inside NZIM is close to zero, at the output of the fishnet metamaterial the phase distribution is conformal to the exit surface, which is planar in our case. Therefore, the curved phase front, propagating from the Soret lens, is transformed into quasi-planar at the interface between the fishnet metamaterial and free space. Due to the inhomogeneity of the fishnet metamaterial, the NZIM regime depends on the number of the plates and tends to deviate significantly when this number is small. This can be clearly seen in Fig 7, where the *E_y_* component on *yz*-plane at 98.75 GHz is plotted for a different number *q* of cascaded plates. With the increase of *q,* the field distribution at the output of the hybrid Soret fishnet metamaterial lens tends to become planar. As a result, the radiation is more directive, i.e. higher directivity and smaller side lobe level. In Fig. 6(d) the numerical results for the gain are shown for an increasing number of plates *q* = 0, 1, 2, 3, 4, 5 (*q* = 0 refers to the case when only the first layer of the lens sandwiched between substrate layers (RO5880™) is present). From the figure, it is evident that increasing the number of plates improves the gain of the hybrid Soret fishnet metamaterial lens. However, a large number results in an increased thickness and therefore higher losses. Hence, for q > 3, the gain decreases. As a compromise solution, we thus designed our lens with the first Soret layer plus 3 fishnet plates. Such design assures lower losses and a maximum gain of metalens antenna.

To conclude, a hybrid Soret fishnet metamaterial lens antenna has been designed, numerically analysed and measured at millimetre-waves. The analysis demonstrates that the application of the metamaterial with a refractive index near zero can improve the radiation characteristics. However, because of the inhomogeneity of the fishnet metamaterial, an optimum number *q* = 3 of stacked fishnet plates has been found. The experimental results show the good performance of the lens antenna and are in good agreement with analytical and simulations results. The proposed design, which is thin, relatively cheap and easy to fabricate, demonstrates a solution to improve the radiation characteristics of the FZPAs. Such compact devices can find applications in wireless systems.

## Methods

### Infinite fishnet index of refraction

The effective refractive index of the infinite fishnet metamaterial was calculated using the eigenmode solver of the commercial software CST Microwave Studio™, using periodic boundaries and assuming perfect electric conductor and lossless dielectric for simplicity. A fine tetrahedral mesh was used, with minimum and maximum edge lengths of 0.009 mm (~0.003λ_0_) and 0.33 mm (~0.1λ_0_), respectively.

### Finite fishnet index of refraction

For the finite fishnet metamaterial model the commercial substrate Rogers RO5880™ was used as a dielectric, with dielectric permittivity ε_r_ = 2.2 and a loss tangent tanδ = 9×10^−4^. The effective refractive index of the fishnet metamaterial, consisting of 4 periods with periodicity *d_z_*, was obtained from the phase of the electric field inside the structure (assuming that the transmittance is high), using the frequency domain solver in the range 90–110 GHz. Unit cell boundary conditions were used and the metal was modelled as aluminum (σ_Al_ = 3.56×10^7^ S/m). The effective index of refraction was calculated as *n_m_* = Δφ/(*k*_0_Δ*d*), where Δφ is the phase variation in the thickness Δ*d* and *k*_0_ is wave number in free-space. A fine tetrahedral mesh is used, with minimum and maximum edge lengths of 0.007 mm (~0.003λ_0_) and 0.64 mm (~0.2λ_0_), respectively.

### Huygens-Fresnel principle

This principle states that any point where a wave front impinges acts as a secondary wave source. For simplicity and given the holey nature of the fishnet, the holes of the first layer of the Soret lens were represented as point sources. Also, reflection and absorption were neglected. Furthermore, the phase front propagation in the fishnet metamaterial was assumed to be planar, therefore the phases of all sources were taken as equal. In addition, all sources were assumed to radiate a vertically polarized (*E_y_*) wave with the same amplitude, equal to the *S_21_* coefficient of the fishnet metamaterial. With this assumptions, the resulting field at each point of space (*x, y, z*) can be calculated by adding the fields of all sources. Mathematically, this can be written as: 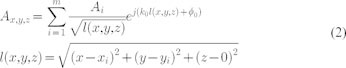
where *A_i_*is amplitude of point source *i*; *l*(*x,y,z*) is the distance between the point source *i* and point in the space (*x,y,z*); *k_0_* is the wave vector in free space; *x_i_*, *y_i_* are the position coordinates of the holes; *φ_0_* is the phase of incident plane wave.

### Focal properties simulations

The numerical study of the metalens was done using the transient solver (finite-integration time-domain method) of CST Microwave Studio™. The metal aluminum layer was modelled as a lossy metal with the bulk conductivity of aluminum (σ_Al_ = 3.56×107 S/m). A fine hexahedral mesh was used with minimum and maximum mesh cell sizes of 0.088 mm (~0.03λ_0_) and 0.25 mm (~0.08λ_0_), respectively. The metalens was illuminated by a vertically polarized (*E_y_*) plane wave impinging normally on the fishnet side. Perfectly matched layers (i.e., the solver-defined *open add space* boundaries) were used at the boundaries of the simulation box to emulate a lens in free space. Given the two-fold symmetry of the problem, electric and magnetic symmetries were imposed in the *xz*-plane (*y* = 0) and *yz*-plane (*x* = 0), respectively, to reduce computation time. The simulation was run for a sufficiently long time to ensure steady-state regime so that the continuous-wave information computed by Fourier transformations was valid. The colour-map of the power spectrum as a function of z position was obtained by placing E-field and H-field probes along the optical axis (*z*-axis) with a 0.05 mm step. These probes record the waveform at their positions and by Fourier transformation, the E- and H-field spectra are obtained.

### Focal properties measurement

The Soret fishnet metalens was fabricated using aluminum foil with thickness 0.021±0.003 mm perforated and cut by laser. The substrate Rogers RO5880™ with permittivity ε*_r_* = 2.2 and thickness 0.381 mm was used as dielectric spacer between successive aluminum foils and as additional protective outer layers. The experimental characterization was done using an AB Millimetre™ quasi-optical vector network analyzer with the setup illustrated in Fig 4. To illuminate the lens, a W-band corrugated horn antenna was placed at a distance *L* = 4000 mm from the lens. At this distance the radius beam waist of the Gaussian beam is ~400 mm, which ensures a uniform illumination of the lens. A waveguide probe WR-8.0 was used as a detector, for the *xz* raster scanning. Millimeter-wave absorbers were used throughout the setup to mimic anechoic chamber conditions. The calibration was done by recording the transmitted power without the lens. For the power distribution as a function of frequency and *z* position, the lens was placed in the setup and the detector was moved from 0.5 to 10 mm away from the lens along *z*-axis (with 0.05 mm steps) while recording the spectrum in the range 90–110 GHz.

### Radiation diagram analysis

For the radiation characteristic analysis the realistic waveguide probe WR-8.0 was used as a feeder and placed at the previously numerically-found position *z* = 1.9 mm, which corresponds to the maximum radiation of the FZPA. The rest of the simulation parameters were described previously. Far-field monitors were used to record the radiation pattern of the lens within the frequency range 90–110 GHz with a step of 0.25 GHz

In the measurement, the waveguide probe WR-8.0 was used as a feeder placed at the experimental focal length *FL* = 1.9 mm (0.63λ_0_). A schematic of the experimental setup along with the fabricated Soret fishnet metamaterial lens is shown in Fig. 4. Meanwhile, a high gain standard horn antenna was placed 4000 mm away from the flat face of the zoned lens to detect the radiated power. To measure the angular distribution of the radiated power, the feeder and the zoned lens stood on a rotating platform that can rotate from −90 deg to +90 deg with 0.5 deg step. Absorbing material was also used throughout the setup for reflection suppression.

In order to obtain the gain of the lens antenna, the received power was measured using a standard gain horn antenna with the lens and waveguide probe as a transmitter. Then the lens was removed and a standard high gain horn antenna replaced the transmitter. In the numerical simulations the realized gain was directly calculated by the built-in far-field monitors of CST Microwave Studio™.

## Author Contributions

B.O, M.B. and M.N.-C. conceived the idea and supervised the study. G.C. fabricated the sample. B.O. and J.T. performed the experiment and data analysis. B.O. and V.P.-P. contributed to the analytical and numerical results. B.O. and M.N.-C. wrote the manuscript. All authors participated in the discussion and manuscript preparation.

## Figures and Tables

**Table 1:** Focal properties and radiation pattern parameters

	Frequency, GHz	*FL^1^*, mm	*FWHM^2^*, mm		*DF^3^*, mm	HPBW^4^*,* deg		FNBW^5^***,*** deg		FSLL^6^***,*** deg		Directivity, dBi
		*E*-plane	*E*-plane	*H-*plane	*E*-plane	*E*-plane	*H-*plane	*E*-plane	*H-*plane	*E*-plane	*H-*plane	
		*H-*plane			*H-*plane							
Analytical	95	1.87	1.13	1.58	1.45	-	-	-	-	-	-	-
	λ*_0_* = 3.15 mm	0.59λ_0_	0.36λ_0_	0.5λ_0_	0.46λ_0_							
Simulation	98.75	1.94	1.39	1.58	1.97	8.1	12.7	48	50	−13.1	−9.9	26.0
	λ*_0_* = 3.04 mm	0.64λ_0_	0.46λ_0_	0.52λ_0_	0.65λ_0_							
Experimental	96.45	1.9	1.44	1.73	2	18.1	13.7	42	67	−13.5	−20.7	22.2
	λ*_0_* = 3.15 mm	0.61λ_0_	0.46λ_0_	0.56λ_0_	0.64λ_0_							

1FL is the focal length.

2FWHM is the full width at half maximum.

3DF is the depth of focus.

4HPBW is the half-power beam width

5FNBW is the first null beam width

6FSLL is the first side-lobe level
